# Supplementary material for: An open-source framework for large-scale, flexible evaluation of biomedical text mining systems
Source: J Biomed Discov Collab. 2008 Jan 29;3:1. doi: 10.1186/1747-5333-3-1 (PMC2276192; doi:10.1186/1747-5333-3-1)
Supplement: Additional file 1 — Evaluation platform source code. The additional file contains the software evaluation framework discussed in this paper. The most current release can be downloaded from SourceForge.net [1]. See the accompanying README file for instructions on installation and use. Also included in the distribution is a collection of UIMA wrappers for some commonly used BioNLP tools and annotated corpora. [file 1747-5333-3-1-S1.zip › bionlp_uima_repository-1.0.1/docs/api/src-html/edu/uchsc/ccp/uima/util/opendmap/AnnotationMap.html]

```
001    /**
002     * The OpenDMAP for Protege Project
003     * February 2006
004     */
005    package edu.uchsc.ccp.uima.util.opendmap;
006    
007    import java.io.File;
008    import java.io.IOException;
009    import java.io.PrintStream;
010    import java.util.HashMap;
011    import java.util.Vector;
012    
013    import javax.xml.parsers.DocumentBuilder;
014    import javax.xml.parsers.DocumentBuilderFactory;
015    import javax.xml.parsers.ParserConfigurationException;
016    
017    import org.w3c.dom.Document;
018    import org.w3c.dom.Element;
019    import org.w3c.dom.NamedNodeMap;
020    import org.w3c.dom.Node;
021    import org.w3c.dom.NodeList;
022    import org.xml.sax.SAXException;
023    
024    import edu.uchsc.ccp.opendmap.DMAPItem;
025    import edu.uchsc.ccp.opendmap.Reference;
026    
027    /**
028     * This class loads a translation map for use in moving from
029     * CAS annotations to OpenDMAP input tokens and from OpenDMAP
030     * output references to CAS annotations.
031     * 
032     * @author R. James Firby
033     */
034    public class AnnotationMap {
035            
036            /* The set of CAS annotations to use as input tokens and the slots to include */
037            private HashMap<String, String> inputMap = null;
038            private HashMap<String, HashMap<String, String>> inputSlotMap = null;
039            
040            /* The set of output references to turn into CAS annotations and the slots to include */
041            private HashMap<String, String> outputMap = null;
042            private HashMap<String, HashMap<String, String>> outputSlotMap = null;
043    
044            /* */
045            private HashMap<String, String> referenceRoots = null;
046            /* Cached reference types of interest from the outputMap */
047            private Vector<String> referencesOfInterest = null;
048            
049            /**
050             * Create an empty annotation map.  Mappings will need to be added explicitly.
051             */
052            public AnnotationMap() {}
053    
054            /**
055             * Create an annotation map from an XML file.
056             * 
057             * @param filename The name of the file holding the configuration.
058             * @throws ParserConfigurationException If there is no good XML parser available
059             * @throws SAXException If there is an XML syntax problem in the configuration file
060             * @throws IOException If there is a problem reading the file
061             */
062            public AnnotationMap(String filename) throws ParserConfigurationException, SAXException, IOException {
063                    // Turn the filename into a File
064                    File file = new File(filename);
065                    Document document = readConfigurationFile(file);
066                    // Process the input annotation map
067                    NodeList nodes = document.getElementsByTagName("input-annotation");
068                    buildInputAnnotationMap(nodes, file);
069                    // Process the output annotation map
070                    nodes = document.getElementsByTagName("output-annotation");
071                    buildOutputAnnotationMap(nodes, file);
072            }
073            
074            /**
075             * Check whether any CAS annotations other than TokenAnnotations are to be 
076             * used as input tokens (typically named entities).
077             * 
078             * @return 'True' if there is at least one CAS annotation type to use
079             */
080            public boolean hasInputMentionsOfInterest() {
081                    return ((inputMap != null) && (inputMap.size() > 0));
082            }
083            
084            /**
085             * Check whether a specific CAS annotation mention is to become an input
086             * token.
087             * 
088             * @param name The name of the CAS annotation mention
089             * @return 'True' if this type of annotation mention should become a token
090             */
091            public boolean isInputMentionOfInterest(String name) {
092                    if ((inputMap == null) || (name == null)) {
093                            return false;
094                    } else {
095                            return inputMap.containsKey(canonicalize(name));
096                    }
097            }
098            
099            /**
100             * Get the type of DMAP Protege Frame ti use for the input token corresponding
101             * to this type of CAS annotation mention.
102             * 
103             * @param name The name of the CAS annotation mention
104             * @return The DMAP Protege Frame type to represent this annotation
105             */
106            public String getInputMentionReferenceType(String name) {
107                    if ((inputMap == null) || (name == null)) {
108                            return null;
109                    } else {
110                            return inputMap.get(canonicalize(name));
111                    }               
112            }
113            
114            /**
115             * Set the type of DMAP Protege Frame to use for the input token corresponding
116             * to this type of CAS annotation mention.
117             * 
118             * @param name The name of the CAS annotation mention
119             * @param dmapType The DMAP Protege Frame type to represent this annotation
120             */
121            public void setInputMentionReferenceType(String name, String dmapType) {
122                    if ((name != null) && (dmapType != null)) {
123                            if (inputMap == null) {
124                                    inputMap = new HashMap<String, String>();
125                            }
126                            inputMap.put(canonicalize(name), dmapType);
127                    }
128            }
129            
130            /**
131             * Check whether a particular slot of a CAS annotation mention should be included
132             * in the DMAP Protege Frame used for the input token.
133             * <p>
134             * By default, no slots are included.
135             * 
136             * @param mention The CAS annotation mention name
137             * @param slot The CAS annotation mention slot name
138             * @return 'True' if this slot should be carried into the DMAP input token
139             */
140            public boolean isInputMentionSlotIncluded(String mention, String slot) {
141                    if ((inputSlotMap == null) || (mention == null) || (slot == null)) {
142                            return false;
143                    } else {
144                            String ccls = canonicalize(mention);
145                            if (!inputSlotMap.containsKey(ccls)) return false;
146                            HashMap<String, String> slotMap = inputSlotMap.get(ccls);
147                            if (slotMap == null) return false;
148                            if (!slotMap.containsKey(slot)) return false;
149                            String dmapName = slotMap.get(slot);
150                            return (dmapName != null);
151                    }
152            }
153            
154            /**
155             * Get the slot name to use in the DMAP reference built to represent this CAS
156             * annotation mention slot.
157             * 
158             * @param mention The CAS annotation mention name
159             * @param slot The CAS annotation mention slot name
160             * @return The DMAP reference slot name to use in the input token for this CAS mention
161             */
162            public String getInputMentionSlotReferenceType(String mention, String slot) {
163                    if ((mention == null) || (slot == null)) return null;
164                    String ccls = canonicalize(mention);
165                    if (inputSlotMap == null) return slot;
166                    if (!inputSlotMap.containsKey(ccls)) return slot;
167                    HashMap<String, String> slotMap = inputSlotMap.get(ccls);
168                    if (slotMap == null) return slot;
169                    String dmapName = slotMap.get(slot);
170                    if (dmapName == null) return slot;
171                    return dmapName;
172            }
173            
174            /**
175             * Set whether or not a CAS annotation mention slot should be included in
176             * DMAP input token references.
177             * 
178             * @param mentionThe CAS annotation mention name.
179             * @param slot The CAS annotation mention slot name.
180             * @param dmapSlot The DMAP reference name for the corresponding slot.  Or null if this slot should be excluded.
181             */
182            public void setInputMentionSlotReferenceType(String mention, String slot, String dmapSlot) {
183                    if ((mention != null) && (slot != null)) {
184                            // Make sure there is a place for this entry
185                            if (inputSlotMap == null) 
186                                    inputSlotMap = new HashMap<String, HashMap<String, String>>();
187                            String ccls = canonicalize(mention);
188                            if (!inputSlotMap.containsKey(ccls)) 
189                                    inputSlotMap.put(ccls, new HashMap<String, String>());
190                            // Get the slot map for this mention type
191                            HashMap<String, String> slotMap = inputSlotMap.get(canonicalize(mention));
192                            slotMap.put(slot, null);
193                    }
194            }
195            
196            /**
197             * Check whether any DMAP output references are to be written back into the CAS.
198             * 
199             * @return 'True' if there is at least one output reference of interest.
200             */
201            public boolean hasOutputReferencesOfInterest() {
202                    return ((outputMap != null) && (outputMap.size() > 0));
203            }
204            
205            /**
206             * Check whether a specific DMAP output reference type should be written
207             * back into the CAS.
208             * 
209             * @param name The name of the DMAP Reference frame type
210             * @return 'True' if this reference should be written back
211             */
212            public boolean isOutputReferenceOfInterest(String name) {
213                    if ((outputMap == null) || (name == null)) {
214                            return false;
215                    } else {
216                            return outputMap.containsKey(canonicalize(name));
217                    }
218            }
219            
220            /**
221             * Check whether a specific DMAP output reference should be written
222             * back into the CAS.
223             * 
224             * @param reference The reference to check
225             * @return 'True' if this reference should be written into the CAS
226             */
227            public boolean isOutputReferenceOfInterest(Reference reference) {
228                    if ((referencesOfInterest == null) || (reference == null)) {
229                            return false;
230                    } else {
231                            for (String cls: referencesOfInterest) {
232                                    if (reference.getItem().isa(cls)) return true;
233                            }
234                            return false;
235                    }
236            }
237            
238            /**
239             * Get the type of CAS annotation mention that should be created to
240             * represent this DMAP reference.
241             * 
242             * @param reference The reference to check
243             * @return The type of CAS annotation mention to create
244             */
245            public String getOutputReferenceMentionType(Reference reference) {
246                    if (reference == null) return null;
247                    String dmapType = reference.getText();
248                    if ((referencesOfInterest == null) || (outputMap == null)) {
249                            return dmapType;
250                    } else {
251                            for (String cls: referencesOfInterest) {
252                                    if (reference.getItem().isa(cls)) {
253                                            String ccls = canonicalize(cls);
254                                            if (outputMap.containsKey(ccls)) {
255                                                    return outputMap.get(ccls);
256                                            } else {
257                                                    return dmapType;
258                                            }
259                                    }
260                            }
261                            return dmapType;
262                    }
263            }
264            
265            /**
266             * Check whether this reference slot should be included in the
267             * annotation mention written back to the CAS.
268             * <p>
269             * By default, all slots are included (the opposite of input
270             * annotation to reference mappings).
271             * 
272             * @param reference The reference to be written back
273             * @param slot The name of the reference slot
274             * @return 'True' if this slot should be included in the CAS mention
275             */
276            public boolean isOutputReferenceSlotOfInterest(Reference reference, DMAPItem slot) {
277                    if ((referencesOfInterest == null) || (reference == null) || (slot == null)) {
278                            return false;
279                    } else {
280                            for (String cls: referencesOfInterest) {
281                                    if (reference.getItem().isa(cls)) {
282                                            String ccls = canonicalize(cls);
283                                            if (!outputSlotMap.containsKey(ccls)) return true;
284                                            HashMap<String, String> slotMap = outputSlotMap.get(ccls);
285                                            if (slotMap == null) return true;
286                                            for (String key: slotMap.keySet()) {
287                                                    if (slot.isa(key)) {
288                                                            String mention = slotMap.get(key);
289                                                            return (mention != null);
290                                                    }
291                                            }
292                                            return true;
293                                    }
294                            }
295                            return false;
296                    }
297            }
298            
299            /**
300             * Get the name to use for this reference slot in the CAS annotation mention
301             * being written back to the CAS.
302             * 
303             * @param reference The reference being written back
304             * @param slot The name of the reference slot
305             * @return The name to use for the corresponding CAS slot
306             */
307            public String getOutputReferenceSlotMentionType(Reference reference, DMAPItem slot) {
308                    if (slot == null) return null;
309                    String slotName = slot.getText();
310                    if ((referencesOfInterest == null) || (reference == null)) {
311                            return slotName;
312                    } else {
313                            for (String cls: referencesOfInterest) {
314                                    if (reference.getItem().isa(cls)) {
315                                            String ccls = canonicalize(cls);
316                                            if (!outputSlotMap.containsKey(ccls)) return slotName;
317                                            HashMap<String, String> slotMap = outputSlotMap.get(ccls);
318                                            if (slotMap == null) return slotName;
319                                            for (String key: slotMap.keySet()) {
320                                                    if (slot.isa(key)) {
321                                                            String mention = slotMap.get(key);
322                                                            if (mention == null) {
323                                                                    return slotName;
324                                                            } else {
325                                                                    return mention;
326                                                            }
327                                                    }
328                                            }
329                                            return slotName;
330                                    }
331                            }
332                            return slotName;
333                    }               
334            }
335            
336            /**
337             * Get the slot filler designated as the root for this reference.  If there
338             * is no designated slot, then just return the reference.
339             * <p>
340             * The root of a reference is the slot value that anchors the reference in
341             * the parsed text and disambiguates the reference from other references of
342             * the same type that were found.  For example, a slot that holds the "verb" for
343             * a reference might be a natural root.
344             * 
345             * @param reference The reference to find the root of.
346             * @return The root for that reference
347             */
348            public Reference getOutputReferenceRoot(Reference reference) {
349                    if ((referencesOfInterest == null) || (reference == null)) {
350                            return reference;
351                    } else {
352                            for (String cls: referencesOfInterest) {
353                                    if (reference.getItem().isa(cls)) {
354                                            String ccls = canonicalize(cls);
355                                            if (!referenceRoots.containsKey(ccls)) return reference;
356                                            String rootSlotName = referenceRoots.get(ccls);
357                                            if (rootSlotName == null) return reference;
358                                            Reference root = reference.getSlotValue(rootSlotName);
359                                            if (root == null) return reference;
360                                            return root;
361                                    }
362                            }
363                            return reference;
364                    }
365            }
366            
367            /**
368             * Get all of the types of the references of interest.
369             * 
370             * @return The names of the reference types of interest.
371             */
372            public Vector<String> getReferencesOfInterest() {
373                    return referencesOfInterest;
374            }
375            
376            /**
377             * Get the type of CAS annotation mention that should be created to
378             * represent this DMAP reference type.
379             * 
380             * @param name The name of the DMAP reference type of interest.
381             * @return The corresponding CAS annotation mention type to create.
382             */
383            public String getOutputReferenceMentionType(String name) {
384                    if ((outputMap == null) || (name == null)) {
385                            return null;
386                    } else {
387                            return outputMap.get(canonicalize(name));
388                    }               
389            }
390            
391            /**
392             * Set the type of CAS annotation mention that should be created to
393             * represent this DMAP reference type.
394             * 
395             * @param dmapType The DMAP reference type of interest.
396             * @param mention The CAS annotation mention type to create for this DMAP type.
397             */
398            public void setOutputReferenceMentionType(String dmapType, String mention) {
399                    if ((mention != null) && (dmapType != null)) {
400                            if (outputMap == null) {
401                                    outputMap = new HashMap<String, String>();
402                            }
403                            String name = canonicalize(dmapType);
404                            outputMap.put(name, canonicalize(mention));
405                            if (referencesOfInterest == null) {
406                                    referencesOfInterest = new Vector<String>();
407                            }
408                            if (!referencesOfInterest.contains(name)) referencesOfInterest.add(dmapType);
409                    }
410            }
411            
412            /**
413             * Get the name of the slot holding the root reference for references of
414             * this DMAP type.
415             * 
416             * @param cls The type of the DMAP reference.
417             * @return The name of the DMAP reference slot holding the root for this type of reference.
418             */
419            public String getOutputReferenceRootSlot(String cls) {
420                    if ((referenceRoots == null) || (cls == null)) {
421                            return null;
422                    } else {
423                            return referenceRoots.get(canonicalize(cls));
424                    }
425            }
426            
427            /**
428             * Set the name of the slot holding the root reference for references of
429             * this DMAP type.
430             * 
431             * @param cls The type of the DMAP reference.
432             * @param root The name of the DMAP reference slot holding the root for this type of reference.
433             */
434            public void setOutputReferenceRootSlot(String cls, String root) {
435                    if ((cls != null)) {
436                            if (root != null) {
437                                    if (referenceRoots == null) 
438                                            referenceRoots = new HashMap<String, String>();
439                                    referenceRoots.put(canonicalize(cls), root);
440                            } else {
441                                    if (referenceRoots != null) {
442                                            referenceRoots.remove(canonicalize(cls));
443                                    }
444                            }
445                    }
446            }
447            
448            /**
449             * Check whether an output reference slot should be included in the CAS
450             * annotation mention being written back to the CAS.
451             * 
452             * @param cls The DMAP reference type
453             * @param slot The DMAP reference slot name
454             * @return 'True' if this slot should be excluded from the CAS
455             */
456            public boolean isOutputReferenceSlotExcluded(String cls, String slot) {
457                    if ((outputSlotMap == null) || (cls == null) || (slot == null)) {
458                            return false;
459                    } else {
460                            String ccls = canonicalize(cls);
461                            if (!outputSlotMap.containsKey(ccls)) return false;
462                            HashMap<String, String> slotMap = outputSlotMap.get(ccls);
463                            if (slotMap == null) return false;
464                            if (!slotMap.containsKey(slot)) return false;
465                            String mention = slotMap.get(slot);
466                            return (mention == null);
467                    }
468            }
469            
470            /**
471             * Set whether an output reference slot should be included in the CAS
472             * annotation mention being written back to the CAS.
473             * 
474             * @param cls The DMAP reference type
475             * @param slot The DMAP reference slot name
476             * @param excluded 'True' if this slot should be excluded from the CAS
477             */
478            public void setOutputReferenceSlotExcluded(String cls, String slot, boolean excluded) {
479                    if ((cls != null) && (slot != null)) {
480                            if (excluded) {
481                                    if (outputSlotMap == null) 
482                                            outputSlotMap = new HashMap<String, HashMap<String, String>>();
483                                    String ccls = canonicalize(cls);
484                                    if (!outputSlotMap.containsKey(ccls)) 
485                                            outputSlotMap.put(ccls, new HashMap<String, String>());
486                                    outputSlotMap.put(slot, null);
487                            } else {
488                                    if (outputSlotMap != null) {
489                                            HashMap<String, String> slotMap = outputSlotMap.get(canonicalize(cls));
490                                            if ((slotMap != null) && slotMap.containsKey(slot)) {
491                                                    String mention = slotMap.get(slot);
492                                                    if (mention == null) slotMap.remove(slot);
493                                            }
494                                    }
495                            }
496                    }
497            }
498            
499            /**
500             * Read an annotation map configuration file into this annotation map.
501             * 
502             * @param file The file to load.
503             * @return The XML DOM document representing the contents of the configuration file.
504             * @throws ParserConfigurationException If there is no valid XML DOM parser.
505             * @throws SAXException If there is a syntax error in the configuration file XML.
506             * @throws IOException If there is a problem reading the file.
507             */
508            private Document readConfigurationFile(File file) throws ParserConfigurationException, SAXException, IOException {
509                    DocumentBuilderFactory factory = DocumentBuilderFactory.newInstance();
510                    DocumentBuilder builder = factory.newDocumentBuilder();
511                    return builder.parse(file);
512            }
513            
514            /**
515             * Populate the input annotation map from entries in the configuration file.
516             * 
517             * @param nodes The XML DOM nodes holding input annotation information.
518             * @param file The file holding the definition of these nodes.
519             */
520            private void buildInputAnnotationMap(NodeList nodes, File file) {
521                    inputMap = new HashMap<String, String>();
522                    for (int i=0; i<nodes.getLength(); i++) {
523                            Node node = nodes.item(i);
524                            if (node instanceof Element) {
525                                    String annotationType = getAttributeValue(node, "mention");
526                                    String dmapType = getAttributeValue(node, "frame");
527                                    if ((annotationType != null) && (dmapType != null)) {
528                                            inputMap.put(canonicalize(annotationType), dmapType);
529                                    } else {
530                                            System.err.println("WARNING: input-annotation missing 'mention' or 'frame' attribute in " + file.getAbsolutePath());
531                                    }
532                                    // The slot mappings
533                                    NodeList slots = ((Element)node).getElementsByTagName("slot");
534                                    if ((slots != null) && (slots.getLength() > 0)) {
535                                            if (inputSlotMap == null) inputSlotMap = new HashMap<String, HashMap<String, String>>();
536                                            // Get the slot map for this input annotation
537                                            HashMap<String, String> slotMap = null;
538                                            dmapType = canonicalize(dmapType);
539                                            if (inputSlotMap.containsKey(dmapType)) {
540                                                    slotMap = inputSlotMap.get(dmapType);
541                                            } else {
542                                                    slotMap = new HashMap<String, String>();
543                                                    inputSlotMap.put(dmapType, slotMap);
544                                            }
545                                            for (int j=0; j<slots.getLength(); j++) {
546                                                    // Get the slot attributes
547                                                    Node slot = slots.item(j);
548                                                    String name = getAttributeValue(slot, "name");
549                                                    String mention = getAttributeValue(slot, "mention");
550                                                    String excludeValue = getAttributeValue(slot, "exclude");
551                                                    if ((mention != null)) {
552                                                            // Add this slot to the various maps
553                                                            boolean exclude = false;
554                                                            if (excludeValue != null) {
555                                                                    exclude = Boolean.parseBoolean(excludeValue);
556                                                            }
557                                                            if (exclude) {
558                                                                    slotMap.put(mention, null);
559                                                            } else if (name != null) {
560                                                                    slotMap.put(mention, name);
561                                                            } else {
562                                                                    slotMap.put(mention, mention);
563                                                            }
564                                                    }
565                                            }
566                                    }
567                            }
568                    }
569            }
570            
571            /**
572             * Populate the output annotation map from entries in the configuration file.
573             * 
574             * @param nodes The XML DOM nodes holding output annotation information.
575             * @param file The file holding the definition of these nodes.
576             */
577            private void buildOutputAnnotationMap(NodeList nodes, File file) {
578                    outputMap = new HashMap<String, String>();
579                    outputSlotMap = new HashMap<String, HashMap<String, String>>();
580                    referenceRoots = new HashMap<String, String>();
581                    for (int i=0; i<nodes.getLength(); i++) {
582                            Node node = nodes.item(i);
583                            if (node instanceof Element) {
584                                    // The attributes mapping from DMAP type to CAS type
585                                    String annotationType = getAttributeValue(node, "mention");
586                                    String dmapType = getAttributeValue(node, "frame");
587                                    if ((annotationType != null) && (dmapType != null)) {
588                                            setOutputReferenceMentionType(dmapType, annotationType);
589                                    } else {
590                                            System.err.println("WARNING: output-annotation missing 'mention' or 'frame' attribute in " + file.getAbsolutePath());
591                                    }
592                                    // The slot mappings
593                                    NodeList slots = ((Element)node).getElementsByTagName("slot");
594                                    if (slots != null) {
595                                            // Get the slot map for this
596                                            HashMap<String, String> slotMap = null;
597                                            dmapType = canonicalize(dmapType);
598                                            if (outputSlotMap.containsKey(dmapType)) {
599                                                    slotMap = outputSlotMap.get(dmapType);
600                                            } else {
601                                                    slotMap = new HashMap<String, String>();
602                                                    outputSlotMap.put(dmapType, slotMap);
603                                            }
604                                            for (int j=0; j<slots.getLength(); j++) {
605                                                    // Get the slot attributes
606                                                    Node slot = slots.item(j);
607                                                    String name = getAttributeValue(slot, "name");
608                                                    String mention = getAttributeValue(slot, "mention");
609                                                    String rootValue = getAttributeValue(slot, "root");
610                                                    String excludeValue = getAttributeValue(slot, "exclude");
611                                                    if ((name != null)) {
612                                                            // Add this slot to the various maps
613                                                            boolean exclude = false;
614                                                            if (excludeValue != null) {
615                                                                    exclude = Boolean.parseBoolean(excludeValue);
616                                                            }
617                                                            if (exclude) {
618                                                                    slotMap.put(name, null);
619                                                            } else if (mention != null) {
620                                                                    slotMap.put(name, mention);
621                                                            } else {
622                                                                    slotMap.remove(name);
623                                                            }
624                                                            boolean root = false;
625                                                            if (rootValue != null) {
626                                                                    root = Boolean.parseBoolean(rootValue);
627                                                            }
628                                                            if (root) {
629                                                                    referenceRoots.put(dmapType, name);
630                                                            }
631                                                    }
632                                            }
633                                    }
634                            }
635                    }
636            }
637            
638            /**
639             * Get the value of an attribute from a DOM element.
640             * 
641             * @param node The DOM element.
642             * @param name The name of the attribute.
643             * @return The attribute value.
644             */
645            private String getAttributeValue(Node node, String name) {
646                    if (node.hasAttributes()) {
647                            NamedNodeMap attribs = node.getAttributes();
648                            Node value = attribs.getNamedItem(name);
649                            if (value != null) {
650                                    return getNodeText(value);
651                            }
652                    }
653                    return null;
654            }
655            
656            /**
657             * Get the body text of a DOM element.
658             * 
659             * @param node The DOM element.
660             * @return The body text from the element.
661             */
662            private String getNodeText(Node node) {
663                    if (node.getNodeType() == Node.TEXT_NODE) {
664                            return node.getNodeValue();
665                    } else {
666                            NodeList children = node.getChildNodes();
667                            if (children.getLength() > 0) {
668                                    StringBuffer sb = new StringBuffer();
669                                    for (int i=0; i<children.getLength(); i++) {
670                                            String value = getNodeText(children.item(i));
671                                            if ((value != null) && !value.equals("")) {
672                                                    sb.append(value);
673                                            }
674                                    }
675                                    if (sb.length() > 0) {
676                                            return sb.toString();
677                                    }
678                            }
679                    }
680                    return null;
681            }
682            
683            /**
684             * Put a string into a standard form to use as a hash key.
685             * 
686             * @param thing The string to standardize.
687             * @return The standardized version of the string.
688             */
689            private String canonicalize(String thing) {
690                    if (thing == null) return null;
691                    return thing.trim().toLowerCase();
692            }
693            
694            /**
695             * Print out the contents of this annotation map in a human-readable format.
696             * 
697             * @param stream The stream to print to.
698             * @param indent An indent amount.
699             */
700            public void print(PrintStream stream, int indent) {
701                    printString(stream, indent, "Input Annotation Map");
702                    if ((inputMap == null) || inputMap.isEmpty()) {
703                            printString(stream, indent+2, "Empty");
704                    } else {
705                            for (String key: inputMap.keySet()) {
706                                    printString(stream, indent+2, key + " -> " + inputMap.get(key));
707                            }
708                    }
709                    printString(stream, indent, "Output Annotation Map");
710                    if ((outputMap == null) || outputMap.isEmpty()) {
711                            printString(stream, indent+2, "Empty");
712                    } else {
713                            for (String key: outputMap.keySet()) {
714                                    printString(stream, indent+2, key + " -> " + outputMap.get(key));
715                                    if (outputSlotMap != null) {
716                                            String root = null;
717                                            if ((referenceRoots != null) && referenceRoots.containsKey(key)) root = referenceRoots.get(key);
718                                            HashMap<String, String> slotMap = outputSlotMap.get(key);
719                                            boolean sawRoot = false;
720                                            if ((slotMap != null)) {
721                                                    for (String slot: slotMap.keySet()) {
722                                                            printIndent(stream, indent+4);
723                                                            stream.print(slot);
724                                                            String mention = slotMap.get(slot);
725                                                            if (mention != null) {
726                                                                    stream.print(" -> ");
727                                                                    stream.print(mention);
728                                                            }
729                                                            if (slot.equalsIgnoreCase(root)) {
730                                                                    stream.print(", root");
731                                                                    sawRoot = true;
732                                                            }
733                                                            if (mention == null) stream.print(", exclude");
734                                                            stream.println();
735                                                    }
736                                            }
737                                            if ((root != null) && !sawRoot) {
738                                                    printString(stream, indent+4, root + ", root");
739                                            }
740                                    }
741                            }
742                    }
743            }
744            
745            /**
746             * Print an indent.
747             * 
748             * @param stream The stream to indent.
749             * @param indent The indent amount.
750             */
751      private void printIndent(PrintStream stream, int indent) {
752                    for (int i=0; i<indent; i++) stream.print(" ");
753      }
754            
755      /**
756       * Print a string with a specified indent.
757       * 
758       * @param stream The stream to print to.
759       * @param indent The indent amount.
760       * @param text The string to print with the indent.
761       */
762            private void printString(PrintStream stream, int indent, String text) {
763                    printIndent(stream, indent);
764                    stream.println(text);
765            }
766            
767    }
```
